# Investigation of bovine ephemeral fever virus transmission by putative dipteran vectors under experimental conditions

**DOI:** 10.1186/s13071-020-04485-5

**Published:** 2020-11-26

**Authors:** Jessica E. Stokes, Karin E. Darpel, Simon Gubbins, Simon Carpenter, María del Mar Fernández de Marco, Luis M. Hernández-Triana, Anthony R. Fooks, Nicholas Johnson, Christopher Sanders

**Affiliations:** 1 The Pirbright Institute, Pirbright, Surrey GU24 0NF UK; 2 Virology Department, Animal and Plant Health Agency, Addlestone, Surrey, KT15 3NB UK; 3 Faculty of Health and Medical Science, University of Surrey, Guildford, Surrey, GU2 7YH UK

**Keywords:** Vector, Arbovirus, Calves, *Culicoides*, *Aedes*, *Culex*

## Abstract

**Background:**

Bovine ephemeral fever virus (*Rhabdoviridae*: *Ephemerovirus*) (BEFV) causes bovine ephemeral fever (BEF), an economically important disease of cattle and water buffalo. Outbreaks of BEF in Africa, Australia, Asia and the Middle East are characterized by high rates of morbidity and highly efficient transmission between cattle hosts. Despite this, the vectors of BEFV remain poorly defined.

**Methods:**

Colony lines of biting midges (*Culicoides sonorensis*) and mosquitoes (*Aedes aegypti*, *Culex pipiens* and *Culex quinquefasciatus*) were infected with a strain of BEFV originating from Israel by feeding on blood–virus suspensions and by intrathoracic inoculation. In addition,* in vivo* transmission of BEFV was also assessed by allowing *C. sonorensis* inoculated by the intrathoracic route to feed on male 6 month-old Holstein-Friesian calves**.**

**Results:**

There was no evidence of BEFV replication within mosquitoes fed on blood/virus suspensions for mosquitoes of any species tested for each of the three colony lines. In 170 *C. sonorensis* fed on the blood/virus suspension, BEFV RNA was detected in the bodies of 13 individuals and in the heads of two individuals, indicative of fully disseminated infections and an oral susceptibility rate of 1.2%. BEFV RNA replication was further demonstrated in all *C. sonorensis* that were inoculated by the intrathoracic route with virus after 5, 6 or 7 days post-infection. Despite this, transmission of BEFV could not be demonstrated when infected *C. sonorensis* were allowed to feed on calves.

**Conclusions:**

No evidence for infection or dissemination of BEFV (bovine/Israel/2005-6) in mosquitoes of three different species was found. Evidence was found for infection of *C. sonorensis* by the oral route. However, attempts to transmit BEFV to calves from infected *C. sonorensis* failed. These results highlight the challenge of defining the natural vector of BEFV and of establishing an* in vivo* transmission model. The results are discussed with reference to the translation of laboratory-based studies to inference of vector competence in the field.

**Graphic abstract:**

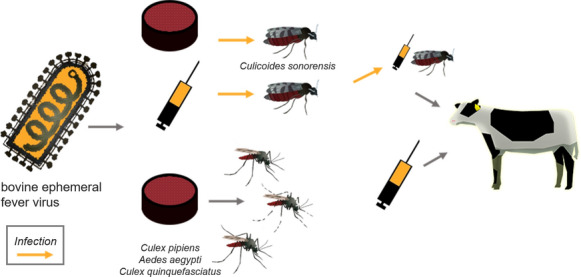

## Background

Bovine ephemeral fever virus (BEFV) is an ephemeroviru*s* within the family *Rhabdoviridae*. BEFV is the causative agent of bovine ephemeral fever (BEF), also known as ‘3 day sickness’, a non-contagious disease of acute febrile clinical signs in cattle and water buffalo. Clinical disease is usually mild in younger animals, often including bi-phasic fever, salivation, lameness and muscle stiffness [1, 2]. However, more severe disease manifestations, such as general depression, muscle weakness, lameness and limp paralysis progressing to sternal recumbency, has been reported, especially in adult dairy cows [3]. Morbidity rates recorded during outbreaks are typically high, in direct contrast to low mortality rates (< 1%) [4]. However, recent outbreaks of BEFV in the Middle East and East Asia have reported higher mortality rates [5–9]. In dairy cows, the economic costs of BEF result from reduced milk yields, loss of valuable cows and/or associated supportive treatment, and in beef cattle, they are due to loss of condition and trade restrictions [1].

BEFV has been recorded in Africa, Asia and Australia, where seasonal outbreaks, particularly following periods of high rainfall, are indicative of vector-borne transmission [5, 10–13]. The risk of BEFV emergence and spread in Europe, however, is challenging to estimate, not least because of the uncertainty concerning the range of vector groups involved in transmission. There is also uncertainty around the potential impact an emergence of BEFV would have given the highly intensive approaches to dairy and beef production in Europe coupled with the lack of previous exposure to the virus. In addition, the unprecedented epizootics of other vector-borne diseases of ruminants, including bluetongue virus (BTV) and Schmallenberg virus, experienced in Europe during the past two decades indicate that arboviruses can emerge in the region without warning, through incursion routes that remain poorly defined [14].

Studies attempting to implicate vectors in the transmission of BEFV can be divided into those detecting the virus in populations of arthropods collected from the field and those involving infections carried out in the laboratory. Evidence of a *Culicoides* vector was first suggested by the isolation and subsequent neutralization test of BEFV from a pool of undifferentiated *Culicoides* species collected in Kenya in 1971 [15]. BEFV has subsequently been isolated from several species of field-caught *Culicoides*. The first isolation of BEFV from *Culicoides* in Australia was from a pool of 100 field-caught *Culicoides brevitarsis* Kieffer 1917 that had been inoculated into mosquitoes through the intrathoracic route and then grown in cell culture [16]. BEFV has also been isolated in Zimbabwe from pooled field-caught *Culicoides imicola* Kieffer 1913 in embryonated eggs and suckling mice, and in *Culicoides coarctatus* Clastrier & Wirth 1961 by direct inoculation into suckling mice [17]. More recently, BEFV RNA has been detected by real time (RT)-PCR in one (out of 166) field-caught *Culicoides arakawae* (Arakawa 1910) in Korea [18].

BEFV has also been isolated from mosquitoes collected during outbreaks in Australia. A mixed pool of culicine mosquitoes [four *Culex* spp,. four *Uranotaenia nivipes* Theobald 1905, one *Uranotaenia albescens* Taylor 1914 and one *Verrallina carmenti* (Edwards 1924)] collected near clinical cases of BEFV led to the isolation of virus on the fourth mouse-brain passage, confirmed by a complement fixation reaction [19]. BEFV has also been isolated from field-caught *Anopheles bancroftii* Giles 1902 on three separate occasions: (i) from a pool of 77 individuals and passaged in mice (intracerebral inoculation) [19]; (ii) from a pool of 50 individuals also following passage in mouse brains, although isolation of virus from the same sample in BHK21 cells was not achieved [20]; (iii) from four pooled *An. bancroftii* after intrathoracic inoculation and passage of the homogenate through live *Aedes aegypti* (Linnaeus 1762), followed by isolation in BHK21 cells [16]. These studies and the correlation of mosquito populations with the spatial and temporal distribution of BEFV led to a hypothesis that transmission was due to a mosquito vector in Australia [16, 19–22]. However, due to the small number of isolations from both *Culicoides* and mosquito species, a definitive identification of a BEFV vector group, or groups, is difficult to ascertain based on field collections of insects alone.

Few studies have attempted to establish the susceptibility of putative vector populations to infection with BEFV in the laboratory. BEFV was successfully isolated 12 days after artificial feeding on a blood–virus suspension from three *Culex annulirostris* Skuse 1889 (out of 23 individuals) and 10 days after membrane feeding on sucrose and BEFV-infected mouse brain from one *C. brevitarsis* (out of 526) and *Culicoides marksi* Lee & Reye 1953 (unknown number of individuals tested, and the number of isolations were not reported) [23]. After feeding on virus–cattle blood and 7–10 days of incubation, evidence of BEFV multiplication in *Cx. annulirostris* was reported (up to 10 individuals/unknown total allowed to feed), but no virus was detected in *Aedes vigilax* (Skuse 1889) [24]. More recently, a study of > 2000 mixed *Culicoides* species fed on a blood–virus suspension in South Africa was unable to isolate virus in any species after 10 days by titration in BHK cells, despite detection of the virus immediately after feeding in 18.9% of individuals tested [25]. Using intrathoracic inoculation of virus suspension, which bypasses midgut barriers to infection in hematophagous flies, it was demonstrated that 70% of *Cx. annulirostris* individuals inoculated with Australian BEFV reference strain BB7721 excreted virus in saliva 7 days after inoculation, although presentation of the methodological details of this experiment was limited (Muller, personal communication in [26]).

Finally, a recent approach that predicted the likely arthropod vector from phylogenetic analysis of viral genome sequences suggested that BEFV was likely to be transmitted by *Culicoides* biting midges, with less support for a mosquito vector [27]. This report aligns with observations of BEFV spread in Kenya, where disease occurrences were observed outside the range of any one mosquito species, but within those of *Culicoides* [11], and with evolutionary analysis of BEFV determining the Australian isolates to be phylogenetically distinct [28]. The results also suggest that geography may influence the vector group responsible for transmission, with greater evidence for the involvement of *Culicoides* in Africa, and for mosquito vectors in Australia, with species of either family being more important in different locations.

Studies of BEFV in cattle have relied on the intravenous injection of blood collected from naturally infected febrile animals [2, 29, 30]. This is not suitable for standardized, controlled infection and transmission studies, particularly due to the transient nature of the disease and potential for undetected co-infections within febrile animals, as well as ethical considerations, cost and virus containment [31]. Limited success has been reported for other infection routes (intramuscular, subcutaneous and intradermal) or inoculum types (synovial fluid and nasal washing) [30, 32]. BEFV can be cultured in BHK-21, Vero, *Aedes albopictus* (Skuse 1985) and hamster lung cell lines [1], and this has facilitated genome sequencing [33]. Attempts to infect the bovine host or bovine cell lines with tissue culture-adapted BEFV strains have so far failed to lead to replication [4]. Similarly, strains derived from BEFV-inoculated mouse brain homogenate have also been reported to lack pathogenicity when injected into bovids [29].

Natural models for the transmission of viruses between ruminants and *Culicoides* have been used in various studies to explore the transmission of BTV within a controlled and standardized experimental design [34, 35] that could greatly facilitate BEFV research. In this study, we investigated the susceptibility to infection of one *Culicoides* and three mosquito species for BEFV. We then assessed the potential to develop a more natural *in vivo* transmission model for this virus using the infection of calves under controlled conditions.

## Materials and methods

### Virus

A strain of BEFV was obtained from the Kimron Veterinary Institute, Israel, which had been isolated from a cow (*Bos taurus*) in 2006 in Israel, and its genome fully sequenced [33]. On receipt of this isolate, designated (bovine/Israel/2005-6a), it was passaged once in T175 tissue culture flasks (Greiner Bio-One, Stonehouse, UK) on 80% confluent African green monkey kidney (Vero) cells with Dulbecco’s Modified Eagle Medium (DMEM) (Sigma-Aldrich, Gillingham, UK) supplemented with 1% penicillin/streptomycin (Sigma-Aldrich), with either 5% heat-inactivated fetal calf serum (+ FCS) or without FCS (− FCS) (Thermo Fisher Scientific, Loughborough, UK). Infected cells were incubated at 37 °C, 5% CO_2_ until a 100% cytopathic effect was observed. The cells were then harvested by centrifugation at 1000* g* at 8°C for 10 min and the supernatant collected in 1.5- to 3-ml volumes and stored at − 80 °C. Cell pellets were also collected, re-suspended in 5–7.5 ml DMEM and stored at − 80 °C. The infectious titer of the strain (cultured both with FCS and without FCS) was determined by a tenfold endpoint titration run in 96-well flat-bottom microplates (Thermo Fisher Scientific), in quadruplet, on a monolayer of Vero cells in 100 µl DMEM supplemented with 1% penicillin/streptomycin and 10% FCS; this was expressed as the 50% tissue culture-infective dose (TCID_50_) calculated by the Spearman–Karber method [36]. The infectious titer recorded in Vero cell culture for BEFV + FCS and BEFV − FCS was 7.5 log_10_ TCID_50_/ml and 6.6 log_10_ TCID_50_/ml, respectively.

### Virus detection

#### Detection of BEFV genome by quantitative RT-PCR

Nucleic acid was extracted from 100 µl of whole blood or 100 µl of insect homogenate using the KingFisher™ Flex Purification robotic extraction system (Thermo Fisher Scientific) with the MagMAX CORE Nucleic Acid Purification kit (Thermo Fisher Scientific) according to manufacturer’s instructions. A pair of specific primers and a TaqMan probe were designed targeting the BEFV glycoprotein of the BEFV strain bovine/Israel/2005-6 [33] (Table 1).

**Table 1:** Primers developed to detect bovine ephemeral fever virus

Primer	Sequence	Final concentration	Genome location (BEFV genome MN078236)
BEFV_F	5'-CATTGGGAATGCATTACAGT-3'	10 pmoles/ml	3683–3202
BEFV_P	5'-[6FAM]AGATTATGGGAAGCTCCAGA[TAM ]-3'	5 pmoles/µl	3737–3756
BEFV_R	5'-GTTTGGTTTTCTATACTCCAC-3'	10 pmoles/µl	3838–3858

BEFV, Bovine ephemeral fever virus

The BEFV quantitative RT-PCR (RT-qPCR) was performed using the iTaq™ Universal Probes One-Step kit (Bio-Rad, Watford, UK). A total reaction volume of 20 µl containing 10 µl 2× iTaq universal probe reaction mix, 0.5 µl of BEFV_F forward primer and 0.5 µl BEFV_R reverse primer, 0.5 µl of BEFV_P probe, 0.5 µl iScript advance reverse transcriptase, 7 µl nuclease-free water and 1 µl RNA template. The thermal cycling protocol was run on a Stratagene Fast 7500 machine (Thermo Fisher Scientific) as follows: an initial reverse transcription at 50 °C for 10 min; then polymerase activation and DNA denaturation at 95°C for 5 min, followed by 45 cycles of denaturation at 95°C for 15 s and annealing/extension at 49 °C for 45 s.

### Susceptibility to infection of colony lines

#### Mosquitoes

Groups of *Culex pipiens* Linnaeus 1758 (Caldbeck line, established from field population in 2011, Surrey, UK, The Pirbright Institute [37]), *Culex quinquefasciatus* Say 1823 (TPRI line, from a colony maintained at the Tropical Pesticide Research Institute, Tanzania, reared and maintained at the London School of Hygiene and Tropical Medicine [LSHTM] since 2010 and donated by LSHTM) and *Aedes aegypti* (Biogent line, originally colonized in Cuba, date unknown, donated by Entostudio Srl, Ponte San Nicolò, Italy) were used during the infection experiments. All mosquitoes used were 3- to 7-days-old and starved of sugar for 16–22 h prior to blood feeding, with water removed 3–4 h prior to feeding. The blood meal was composed of defibrinated horse blood (TCS Biosciences Ltd., Botolph Claydon, UK), 1 µM ATP final concentration (Thermo Fisher Scientific) and BEFV at a final titer of 7.7 log_10_ TCID_50_/ml. Groups of > 100 mosquitoes were allowed to feed overnight with the blood meal heated to 37 °C using a Hemotek artificial feeder (Hemotek Ltd., Blackbern, UK). Individuals were then immobilized by FlyNap™ (Carolina Biological Supply, Burlington, NC, USA), and five engorged mosquitoes were sampled at day 0. The remaining mosquitoes were transferred to biopots (www.bugzarre.co.uk) and supplied sugar through honey-impregnated FTA filters (Whatman™, Little Chalfont, UK) and water on partially soaked cotton wool. All mosquitoes were maintained for 14 days at 25 °C. Filters were collected on alternate days up to 14 days post infection. On day 14, all mosquitoes were immobilized with FlyNap, then saliva, legs and bodies were processed separately as previously published [38]. Total RNA was extracted from intact mosquitoes, saliva, legs and bodies and screened for the presence of BEFV using RT-qPCR.

#### *Culicoides sonorensis*

Colony-derived adults of* C. sonorensis* Wirth & Jones 1957 (PIRB -s-3 strain), a BTV vector in North America, were used in the study [39]. Maintenance was as described previously [40], with the exception that the colony was sustained using a Hemotek artificial feeder and horse blood from a commercial supplier (TCS Bioscience, Botolph Claydon, UK). Approximately 500–600 3-day-old adult *C. sonorensis* were exposed to a cattle blood and BEFV tissue culture mixture (1:1; calculated as 6.6 log_10_ TCID_50_/ml BEFV) using a Hemotek reservoir calibrated to 37 °C and a Parafilm™ membrane. After a 30-min exposure, individual* C. sonorensis* were immobilized with CO_2_. Eleven fully blood-fed female* C. sonorensis* were collected immediately after feeding into a sterile 1.5-ml Eppendorf tube and stored at − 80 °C to determine the quantity of BEFV RNA ingested during feeding (day 0). A further 250 fully engorged, blood-fed females were transferred into a cardboard pillbox (Watkins and Doncaster, Leominster, UK), incubated in the dark at 25 °C, 80% relative humidity (RH) and fed daily with 10% sucrose on a cotton pad.

After 8 days of incubation, the surviving *C. sonorensis* were immobilized with CO_2_ and decapitated using sterile 25G hypodermic needles (Thermo Fisher Scientific); the corresponding heads and bodies put into a collection microtube plate (Qiagen, Manchester, UK) with 200 µl of chilled DMEM media and a 3-mm-diameter sterile stainless steel bead. Plates were sealed and then placed in a TissueLyser (Qiagen) as described elsewhere [41]. Nucleic acid was extracted from 100 µl of homogenates using the KingFisher™ Flex Purification robotic extraction system (Thermo Fisher Scientific) with the MagMAX CORE Nucleic Acid Purification kit (Thermo Fisher Scientific) according to the manufacturer’s instructions. Extracted samples were tested individually by RT-qPCR for the BEFV genome. Samples containing BEFV RNA were further investigated by tenfold endpoint titration, run in quadruplet, on Vero cells in 100 µl DMEM supplemented with 1% penicillin/streptomycin and 5% FCS in 96-well flat-bottom microplates. Due to the limited volume of homogenate, the initial dilution was 1:10, giving a detection cut-off of 1.5 log_10_ TCID_50_/ml.

### Intrathoracic inoculation of *C. sonorensis*

Female *C. sonorensis* were intrathoracically (IT) inoculated with virus, thereby bypassing midgut barriers. To determine whether the efficiency of intrathoracic inoculation resulted in a fully disseminated infection, previously reported at 100% for BTV [41], 50 *C. sonorensis* were inoculated with ≤ 0.2 µl 7.5 log_10_ TCID_50_/ml BEFV + FCS using a pulled glass needle and Nanoject II microinjector as described previously [41] and incubated as described for membrane-fed *Culicoides*. After 5 days of incubation, these insects were homogenized, and the nucleic acid extracted and tested for BEFV RNA by PCR as described for above. A further 100 *C. sonorensis* were IT inoculated and then incubated for 6 days, after which they were decapitated, with the heads and bodies of individuals processed as described above to quantify potential BEFV genome dissemination to the head and salivary glands. *Culicoides sonorensis* homogenized immediately after intrathoracic inoculation were processed in the same way to provide a day 0 baseline for the quantity of BEFV genome inoculated into each individual.

### Transmission of BEFV to the bovine host

#### Ethical statement

This animal experiment was carried out in accordance with the UK Animal Scientific Procedure Act (ASPA) 1986 which transposes European Directive 2010/63/EU into UK national law. All animal procedures carried out were reviewed and approved by the Animal Welfare and Ethics Review Board at the Pirbright Institute and conducted in compliance with Project Licence P96CE012 granted by the UK home office.

#### Generation of BEFV IT-inoculated* C. sonorensis* and homogenate inoculum

A total of 885 *C. sonorensis* females were inoculated with 0.2 µl 7.5 log_10_ TCID_50_/ml BEFV + FCS, with 24 individuals immediately stored at − 80°C to act as day 0 controls to determine the initial quantity of BEFV genome. The remaining inoculated individuals were transferred to pillboxes (74−103 individuals/box) and incubated in the dark at 25 °C, 80% RH with daily feedings of 10% sucrose for 6–7 days at which point survivors were allowed to blood feed upon the cattle as described below. After exposure to cattle, blood-fed *C. sonorensis* were collected, immobilized with CO_2_ and decapitated for homogenization as described previously. Infected *Culicoides* homogenates were later processed to provide inoculum for subsequent cattle infection challenge by taking 100 µl from homogenates that had fed on the respective animals to be challenged followed by filtration through a 0.2-µm filter (Sartorius Stedim Biotech GmbH, Goettingen, Germany).

#### Exposure of cattle to BEFV inoculated* Culicoides* 

A group of four male 6-month-old Holstein-Friesian calves (numbered C1, C2, C3 and C4) weighing 105–198 (median 113.8) kg were used in the study. These animals were held in an insect-secure isolation unit at the Pirbright Institute for the duration of the study with* ad libitum* access to water and roughage (hay and straw) throughout the study, supplemented with twice-daily feeding of concentrate. Further cattle-appropriate enrichment (such as brushes, mineral licks and additional explorative food) was routinely provided. Cattle were monitored at least twice daily by experienced animal technicians, supplemented with CCTV monitoring and on-site veterinary care if required. Blood samples (EDTA and serum) were collected daily from the jugular vein of all calves, in addition to recording the body temperatures and a clinical score based on signs of BEFV (Additional file: 1; Additional file: 2). Calf body temperatures were recorded for 4 days prior to exposure to BEFV and used to provide an average baseline from which deviation due to infection could be assessed. Following exposure to BEFV-inoculated *C. sonorensis*, cattle body temperature was taken twice daily (AM and PM) to allow for the detection of sudden and transient changes in body temperature. The IT BEFV-inoculated *C. sonorensis* were allowed to feed on the rumps of the calves, with one pillbox of *Culicoides* held on each rump of each calf for 20 min. Feeding sites were cleaned and shaved prior to insect exposure to ensure that the *Culicoides* were able to make sufficient contact to blood-feed through the pill box netting. Two pillboxes of BEFV-inoculated *C. sonorensis* were allowed to feed on each animal. One calf (number C2) was exposed to inoculated *Culicoides* for a second time 2 days later due to a poor feeding rate observed in the first exposure. The daily EDTA + blood samples from each calf were tested the same day by RT-qPCR for the presence of BEFV genome and RNAemia as described.

#### Exposure of cattle to uninfected* C. sonorensis*

At 7 days post-infection, naïve *C. sonorensis* (3–4 days old) were exposed to the rump of each calf within pillboxes for 20 min. The *C. sonorensis* were then immobilized with CO_2_ and sorted; fully blood-fed individuals were transferred to pillboxes (150 individuals from each animal/box) and incubated at 25°C, 80% RH while fed 10% sucrose daily for 8 days. In addition, 16 individual *C. sonorensis* fed from each calf were frozen immediately after feeding and stored at −80°C to determine the viral intake with the blood meal. After 8 days, surviving *C. sonorensis* were immobilized with CO_2_ and decapitated, along with the previously stored day 0 controls for homogenization, as previously described.

#### Additional BEFV inoculum exposure of cattle and study end

In the event of failure of transmission of BEFV from inoculated *Culicoides* to a calf*,* the calf was challenged both intradermally (< 1 ml inoculated) and subcutaneously (< 2 ml inoculated) 11 days after initial exposure to the IT BEFV-inoculated *C. sonorensis* with either BEFV -FCS inoculum from tissue culture supernatant (calf number C1 and C3) or with pooled homogenate of inoculated *C. sonorensis* as described above (calf number C2 and C4). All animals were euthanized and examined post-mortem 21 days after first exposure to the BEFV-inoculated *C. sonorensis*.

### Serology

A commercially available sandwich enzyme-linked immunosorbent assay (ELISA) was used to determine antibody titer against BEFV in serum according to the manufacturer’s instructions (MyBioSource, San Diego, CA, USA).

### Statistical analysis

For each group of midges (orally fed, IT inoculated and IT inoculated post-cattle-fed) C_q_ values were compared among groups (i.e. day 0, whole insects, heads or bodies) using a Kruskal–Wallis test. If this was significant (*P* < 0.05), groups were compared using pairwise Wilcoxon rank-sum tests (also known as the Mann–Whitney U-test) to identify differences between groups. In these analyses, insects with no C_q_ value were assigned an arbitrary C_q_ of 40.

Vector competence was computed as the proportion of mosquitoes or midges with a fully disseminated infection using a binomial likelihood. The probability of transmission from vector to host (*b*) was estimated from the outcome of the transmission attempts using maximum likelihood methods. The likelihood function is *L*(*b*) = (1 −* b*)^*n*^, where *n* is the number of infected midges feeding on the calves. All analyses were implemented in Matlab (version 2019b; MathWorks, Natick, MA, USA).

## Results

### Oral susceptibility to BEFV infection of colonized dipteran lines

#### Mosquitoes (*Ae. aegypti*,* Cx. quinquefasciatus*,* Cx. pipiens*)

In order to test the ability of mosquitoes to become infected and transmit BEFV, we allowed mosquitoes from three species to feed on a blood meal containing BEFV strain bovine/Israel/2005-6. Groups (*n* = 20) were maintained until 14 days post infection before processing to provide sufficient time for infection, dissemination and transmission to occur. BEFV was detectable by PCR in RNA extracted from intact mosquitoes immediately after blood-feeding (Table 2). However, none of the honey-impregnated filters removed from all mosquito species tested were positive for BEFV RNA by RT-qPCR up to day 14; control filters, with and without honey, with added BEFV were positive (data not shown). No BEFV RNA was recovered from the body, leg or saliva of any individual on day 14 (Table 2). Based on the outcome of oral infections (i.e. no mosquitoes infected out of 20 feeding), the maximum likelihood estimate for the vector competence in all three mosquito species tested is zero (upper 95% confidence limit 9.2%).

**Table 2:** Summary of bovine ephemeral fever virus testing of blood-fed mosquitoes at 14 days post-infection

Mosquito species	C_q_ value at day 0 (*n* = 5)	C_q_ value at 14 days post-infection (*n* = 20)
*Culex pipiens* Caldbeck	26.57 ± 1.54	Not detected
*Culex quinquefasciatus* TPRI	27.11 ± 1.48	Not detected
*Aedes aegypti* Biogent	30.11 ± 0.8	Not detected

Values in table presented as the median ± range

#### *Culicoides sonorensis*

**Fig. 1:**
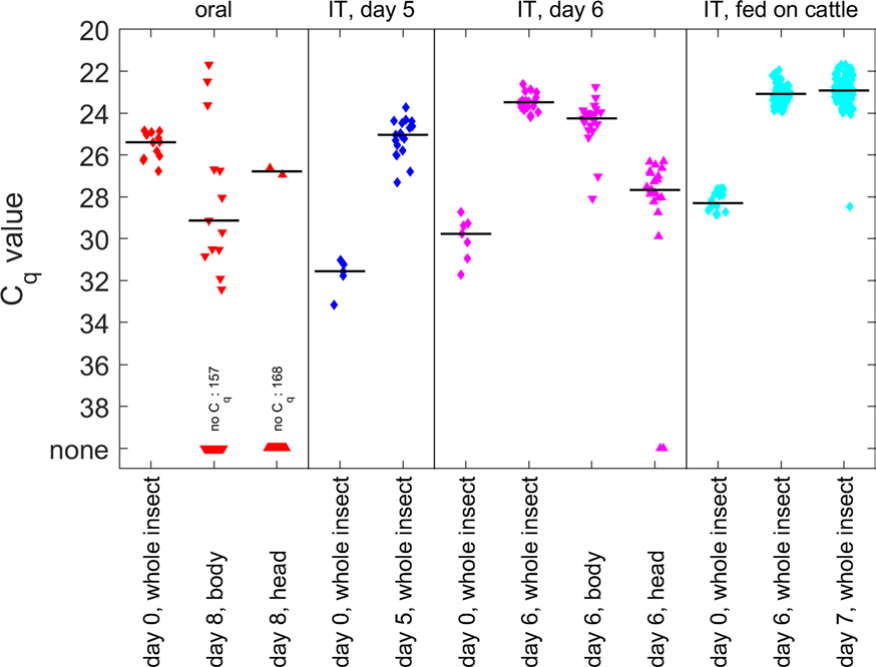
C_q_ values for *Culicoides sonorensis* infected with bovine ephemeral fever virus. Midges were orally fed a blood–virus suspension (oral; red) and incubated for 8 days or intrathoracically inoculated (*IT*) with virus and tested after incubation for 5 (IT, day 5; blue) or 6 days (IT, day 6; magenta) or after 6–7 days incubation and feeding on naïve calves (IT, fed on cattle; cyan). Individuals were processed as whole insects (diamonds) or dissected heads (downward-pointing triangles) or bodies (upward-pointing triangles). Black lines indicate the median value, excluding insects with no C_q_ value

Following the unsuccessful attempt to show infection in mosquitoes, the vector competence of *C. sonorensis* for BEFV vector competence was assessed. A group of 250 *C. sonorensis* were fed BEFV. Eleven individuals were processed for RNA detection by qRT-PCR immediately following feeding on BEFV blood-virus suspension and a median C_q_ of 25.4 (range: 24.8–26.8) was recorded. A total of 204 individuals survived the 8-day incubation period (85.4% survival rate) following feeding on the blood–virus suspension; of these, 170 individuals were tested by RT-qPCR for BEFV RNA. BEFV RNA was detected in 13 of the *C. sonorensis* bodies tested and in two heads (Fig. 1). The 13 *C. sonorensis* produced a median C_q_ value in bodies of 29.1 (range 21.7–32.4), which is significantly higher than the day 0 median (*Z *= − 12.5, *P* < 0.001) (Fig. 1). However, only three bodies had C_q_ values (21.7, 22.5 and 23.6) lower than that of the day 0 median, and the heads of these individuals had *C*_*q*_ values of 26.6 and 26.9 and no *C*_q_ (i.e. BEFV RNA was not detected), respectively. Attempts to titrate the virus from these positive samples, at a detection limit of 1.5 log_10_ TCID_50_/ml, were unsuccessful, with the exception of one *C. sonorensis* body (C_q_ 23.6), for which a BEFV titer of 2.5 log_10_ TCID_50_/ml was recorded.

Using the detection of any BEFV RNA in the head as indicative of a fully disseminated infection (i.e. 2 out of 170 midges), the estimated vector competence was estimated to be 1.2% [95% confidence interval (CI) 0.5–2.4%]. Alternatively, using detection of BEFV RNA in the body at levels above that on day 0 (i.e. 3 of 170 midges), the estimated vector competence was estimated to be 1.8% (95% CI 1.0–2.5%).

### Intrathoracic inoculation of *C. sonorensis*

Based on the low levels of infection observed from the oral infection study, we next used intrathoracic inoculation to increase the number of infected individuals. Of 50 *C. sonorensis* IT inoculated with 0.2 µl BEFV, 19 (38%) survived the 5-day incubation period. The five *C. sonorensis* processed immediately following intrathoracic inoculation were tested for the presence of BEFV by RT-qPCR; median C_q_ was 31.6 (range 31.0–33.2). In comparison, the 19 *C. sonorensis* processed following incubation had a significantly (*Z* = 3.3, *P* < 0.001) lower median C_q_ of 25.0 (range 23.7–27.3) (Fig. 1), indicating an increase in virus genomic RNA with incubation period.

A total of 100 *C. sonorensis* were then IT inoculated with BEFV and incubated for 6 days; of these, 42 (42%) individuals survived the incubation period. The median C_q_ of the seven *C. sonorensis* processed immediately following intrathoracic inoculation was 29.8 (range 28.7–31.7). Of the 42 insects processed following incubation, 21 were processed whole and 21 heads and bodies were processed separately (Fig. 1). BEFV RNA was detected in all bodies (median C_q_ 24.2, range 22.7–28.1) and whole insects (median C_q_ 23.5, range 22.6–24.2). BEFV RNA was also detected in 19 of the 21 tested heads (90.5%; median C_q_ 27.5, range 26.3–29.9). The median C_q_ values differed significantly (χ^2^= 54.3,* df*= 3, *P* < 0.001) among these groups, with the highest C_q_ values (denoting low levels of detected BEFV RNA) obtained in the midges tested at day 0, followed by the heads of insects tested at day 6, then the bodies of insects tested at day 6, with the lowest C_q_ values detected in whole insects tested at day 6 (Fig. 1). These results indicated that intrathoracic inoculation of BEFV led to a productive infection of *C. sonorensis*.

### Transmission of BEFV to the bovine host

In order to maximize the likelihood of onward transmission, 885 *C. sonorensis* were IT inoculated with BEFV before being allowed to feed directly on the calves; of these, 205 were ultimately confirmed to have fed on the calves based on the observation of total or partial engorgement (Table 3). BEFV RNA was detected in all BEFV-inoculated *C. sonorensis* that fed on the calves (day 6: median C_q_ 23.1, range 21.9–23.9; day 7: median C_q_ 22.9, range 21.7–28.4), with C_q_ values that were significantly (*Z *= 6.6, *P* < 0.001) lower (denoting more BEFV RNA) than those in midges tested at day 0 (median C_q_ 28.3, range 27.6–28.8) (Fig. 1). Each calf received bites from at least 35 inoculated *C. sonorensis*, with C_q_ values indicative of fully disseminated BEFV infections.

**Table 3:** Survival and feeding rate of *C. sonorensis* intrathoracically inoculated with bovine ephemeral fever virus (BEFV)

Animal^a^	Total number *C. sonorensis* IT inoculated with BEFV	Number of surviving *C. sonorensis* 6–7 days after inoculation (%)	Total number of fully fed *C. sonorensis*^b^	Total number of partially fed *C. sonorensis*^b^	BEFV *C*_q_ of *C. sonorensis* post-feeding on calves, median (range)^b^
C1	195	65 (33.3)	37	5	23.2 (22.4–28.5)
C2	160	37 (23.1)	13	2	23.2 (22.3–23.3)
C2^b^	152	58 (38.2)	45	5	22.6 (21.7–23.5)
C3	203	66 (32.5)	41	7	23.2 (21.9–23.9)
C4	175	68 (38.9)	43	7	23.2 (22.1–24.0)

IT Intrathoracically

^a^Male 6-month-old Holstein-Friesian calves (numbered C1, C2, C3 and C4)

^b^The number of *C. sonorensis* fully engorged and those partially engorged when fed on each calf is shown, with respective median C_q_ value when tested for BEFV genome by RT-qPCR post feeding

^c^Secondary feeding 2 days later

On study day 7, 7 days after the cattle were initially exposed to *C. sonorensis *by intrathoracic inoculation with BEFV, 600 naïve *C. sonorensis* were allowed to feed on the calves until fully engorged with blood (150 *C. sonorensis* per animal). No BEFV RNA was detectable by RT-qPCR in any insect on day of feeding (16 *C. sonorensis* per animal) or after 8 days of incubation (study day 15).

No clinical signs of viral infection in the calves were observed at any point during the study. Temperatures of all animals remained within ± 1 °C of baseline value across the study. BEFV RNA was not detected within any of the blood samples collected from any calf throughout the study, even after additional BEFV exposure by intradermal/subcutaneous inoculation on study day 11. No antibodies against BEFV were detected by ELISA in serum collected from any calf. Signs of ascites and pericardial fluid were observed at post-mortem; however, no BEFV RNA was detected by RT-qPCR in any of the fluids, and no antibodies were detected by ELISA.

Based on the outcome of the transmission attempts (i.e. no calves affected), the maximum likelihood estimate for the probability of transmission from vector to host was zero (upper 95% confidence limit 0.016).

## Discussion

The aim of this study was to provide evidence for potential vectors of BEFV, particularly those that might transmit the virus to cattle. Experiments that fed artificial blood meals containing BEFV to colony lines of potential dipteran vectors found evidence for replication in *Culicoides* midges (*C. sonorensis*), inferred by comparing the level of viral RNA in incubated flies with that in those killed immediately following blood feeding. The vast majority of insects tested were capable of clearing ingested BEFV to the extent that no viral RNA was detectable, including all of the mosquito species tested (albeit with small sample sizes of 20 individuals for each colony line). In *C. sonorensis*, a total of 170 individuals were tested, of which 13 returned a positive signal for BEFV RNA when their bodies were processed, including three individuals that demonstrated higher viral RNA levels (lower C_q_ value) than those obtained in individuals processed immediately after blood-feeding (day 0 *C. sonorensis*), indicating viral replication. Studies of infection in *C. sonorensis* with other viruses (e.g. BTV) have previously demonstrated infections where there was potential both for retention of virus at low and sub-transmissible levels of infection [35]. This can occur through restriction of the virus to the hind mid-gut due to a mesenteron escape barrier and restriction to the fat body due to a hemocoel dissemination barrier [42]. The evidence of viral RNA present in the heads of two individuals indicates that full dissemination of BEFV to secondary target organs, which is necessary for onwards transmission, did occur, which in previous studies using *C. sonorensis* and BTV has included salivary glands [41]. This result would suggest that *C. sonorensis* has the potential to act as a vector for BEFV, with a low vector competency, in the field despite no overlap in current geographic range of this species and the virus. To further investigate the risk of BEFV transmission within a European context, field-caught *Culicoides* species should be tested for susceptibility to infection through oral exposure to virus.

All *C. sonorensis* that were IT inoculated with BEFV contained RNA at 5, 6 or 7 days post-infection. This technique bypasses the mesenteron infection and escape barriers as well as the hemocoel dissemination barrier and results in full infection, as demonstrated in *C. sonorensis* IT inoculated with BTV. In addition, while *C. sonorensis* infected via this route consistently infect ruminants with BTV with high efficacy [43], this was not observed for BEFV in the present study. The numbers of IT inoculated *Culicoides* fed on each calf would have been more than sufficient to establish infection and viremia in BTV/sheep infection studies [43]. It is possible that infection of the salivary glands did not occur despite virus dissemination to the head. This would be an indication of a salivary gland infection barrier for BEFV, something that has not been reported for any other *Culicoides*-borne virus, but which could be further explored in future studies through the use of honey-impregnated filters or direct saliva collections [44]. It is more likely that the virus, despite replication in the insect vector, was not infectious to the bovine host, as appears to be the case with all infection routes other than the intravenous inoculation of viremic blood.

A reason for this lack of infectivity could be due to *in vitro* cultivation of BEFV from initial isolation leading to the adaptation of BEFV to the cell line used. This has previously been reported for tissue culture-adapted foot and mouth disease virus type O pre-selecting for heparin-binding viruses, which were subsequently attenuated in the natural host [45]. Indeed, the expression of the β and γ open reading frames (ORFs) has been previously reported to be selectively supressed by mutation during BEFV adaption to cell culture (PJ Walker, unpublished data in [4]), with the full genome sequence of this tissue-cultured strain used in this study revealing mutations in the α3 and γ ORFs [33]. The functions of the proteins encoded by these regions are currently unknown, therefore, potentially affecting *in vivo* infection of virus isolates compared to non-tissue adapted virus. It was hoped that infection and replication within the potential *Culicoides* vector might select viral variants still capable of replicating in the ruminant host as an approximation of alternating viral replication between insect and mammalian hosts. Indeed, previous studies determined that the genetic diversity of BTV passaged on mammalian cells was significantly reduced when compared to BTV passaged on insect cell lines (KC cells derived from embryonic *C. sonorensis*) ([46] discussed in [47]).

The successful detection of BEFV genome by RT-qPCR, but the lack of successful virus titration from the original insect material were not surprising considering the potential for losses in infectivity during the freeze–thaw cycles between collection, processing for RT-qPCR and titration, enhanced by an additional freeze–thaw cycle due to the malfunction of the − 80 °C storage freezer between the PCR analyses and the titration experiments. The potential for a loss of infectivity was supported by an observed tenfold drop in infectious virus between titrations of the BEFV, and the impact of repeated freeze–thaw cycles has been previously noted as a reason for a lack of re-isolation of BEFV from original insect material [19].

Published data on the arthropod vectors of BEFV have identified a number of potential vectors, including biting midges of the genus *Culicoides*, and mosquitoes, such as *Culex annulirostris*, found in Australia, parts of Southeast Asia and islands of the western Pacific. Oral infection studies with BEFV did not lead to infection of *Culex pipiens*, *Cx. quinquefasciatus* or *Aedes aegypti* mosquitoes with this virus, but evidence was found for infection in *Culicoides* biting midges. It should be noted that although only a relatively small number of mosquitoes were preliminarily investigated for BEFV infection compared to the number of *Culicoides*, this does reflect field densities of these vectors, where a low vector competence in *Culicoides* may be compensated by the very high local abundance and high biting rate. An estimated competence of < 10% for all the mosquito species tested and absence of evidence of dissemination suggest a low vectorial capacity for BEFV. This result implicates *Culicoides*—rather than mosquitoes—as a likely vector for the virus should it be introduced into Europe, and points to midge control measures as being the most effective to implement.

This study highlights the challenges of carrying out studies on the transmission of BEFV under laboratory conditions. Evidence of replication and dissemination of the bovine/Israel/2005-6 BEFV strain in *C. sonorensis* was demonstrated through both membrane feeding on blood–virus suspension and intrathoracic inoculation. Despite this, an *in vivo* study aimed at establishing a bovid: *C. sonorensis*: BEFV model failed, due to a lack of transmission from what were hypothesized to be fully infected insects into the calves. The reasons for this failure remain undetermined but may relate to previously encountered challenges in working with intensively cell culture-passaged BEFV strains. Future studies will examine dissemination of BEFV within this *Culicoides* species and why onwards transmission to calves did not result in virus replication in the ruminant host despite successful insect blood-feeding and dissemination of the virus in the insect vector.

## Conclusions

To the best of our knowledge, the results of this study demonstrate the replication of BEFV in *C. sonorensis* for the first time, both by feeding through membranes on blood–virus suspensions and through intrathoracic inoculation. No replication was observed in mosquitoes. Attempts to transmit BEFV via *C. sonorensis* feeding on cattle, however, failed, and thus the dissemination of the virus in the insect vector requires further investigation.

## Supplementary information

**Additional file 1**: Animal temperatures and clinical scoring notes.

**Additional file 2**: RT-qPCR and titration results for Culicoides sonorensis orallyfed BEFV and intrathoracically inoculated with BEFV.

## Data Availability

All data generated or analysed during this study are included in this published article and its additional file.
